# Comparative Study of Physicochemical Properties and Starch Granule Structure in Seven Ginkgo Kernel Flours

**DOI:** 10.3390/foods10081721

**Published:** 2021-07-26

**Authors:** Yan Lu, Weizhuo Hao, Xiaomin Zhang, Yue Zhao, Yang Xu, Jixun Luo, Qing Liu, Qiaoquan Liu, Li Wang, Changquan Zhang

**Affiliations:** 1 Key Laboratory of Plant Functional Genomics of the Ministry of Education, Co-Innovation Center for Modern Production Technology of Grain Crops, Agricultural College of Yangzhou University, Yangzhou 225009, China; luyan@yzu.edu.cn (Y.L.); haoweizhuo1@163.com (W.H.); zhaoyue_zyy@163.com (Y.Z.); yangx@yzu.edu.cn (Y.X.); qqliu@yzu.edu.cn (Q.L.); 2 College of Horticulture and Plant Protection, Yangzhou University, Yangzhou 225009, China; xiaominzhang0102@163.com (X.Z.); liwang@yzu.edu.cn (L.W.); 3 CSIRO Agriculture and Food, GPO Box 1600, Canberra, ACT 2601, Australia; jixun.luo@csiro.au (J.L.); Qing.liu@csiro.au (Q.L.)

**Keywords:** *Ginkgo biloba* L., kernel flours, starch, protein, physicochemical properties

## Abstract

*Ginkgo biloba* L. is an important economic tree species in China, and its kernels have been used as a popular food in Asian countries. Herein, the morphology, basic chemical components, starch granule structures, and physicochemical properties of kernel flours from seven ginkgo cultivars were investigated, and their relationships were analyzed. The kernels were oval or spherical in shape, with variable sizes. The starch granules exhibited both regular and irregular Maltese cross patterns. Amylose was mainly distributed in amorphous growth rings. A spatial variation in the 865/942 cm^−1^ ratio was observed within individual starch granules. Variations in total starch content, apparent amylose content (AAC), crude protein content (CPC), total amino acid content (TAAC), starch fine structure, and thermal and pasting properties were observed among the seven kernel flours. Pearson correlation coefficients and principle component analyses showed that the thermal properties were affected by kernel CPC, TAAC, AAC, and starch fine structure, while the pasting properties were affected by AAC and starch fine structure. Furthermore, experiments showed that the seed protein structure and α-amylase activity affected the pasting properties of ginkgo kernel flours.

## 1. Introduction

*Ginkgo biloba* L. is among the most primitive extant gymnosperms and a “living fossil” plant [1]. In China, ginkgo is widely cultivated throughout the temperate and subtropical regions [2,3], where ginkgo seeds have been produced commercially for more than 600 years [4,5]. Ginkgo seeds are covered with a soft and fleshy sarcotesta, a hard mesosperm, a papery endotesta, and a kernel that consists of endosperm and embryo [6]. Owing to its high nutritional value and delicious taste, roasted ginkgo kernels are a popular snack food in some Asian countries, including China, Japan, and Korea [7]. Furthermore, ginkgo seeds have various uses in traditional medicine to treat pulmonary diseases and improve general health [8,9,10]. Today, *G. biloba* L. is among the most important economic tree species in China.

When eating ginkgo kernels as an intact grain, consumers prefer cooked ginkgo kernels, which have a soft and sticky texture. Ginkgo kernels are the main edible part of the seed, containing 60–70% starch, 10–20% protein, 2–4% lipids, 0.8–1.2% pectin, and about 6% sucrose, on a dry weight basis [4,11,12]. Furthermore, ginkgo seeds contain trace elements, vitamins, and abundant secondary metabolites [13,14]. Therefore, the biochemical properties of ginkgo kernels play an important role in determining the quality and functionality of food products derived from ginkgo seeds [15,16]. Studies on the structural characteristics of ginkgo starch have shown that starch granules have A-type, C-type, or C_A_-type crystallinities, with a degree of crystallinity of 27–41%. These granules have an oval or spherical shape with a central hilum, containing high levels of A and B1 chains of amylopectin [17,18,19]. We recently investigated the starch fine structures and physicochemical properties of seven ginkgo cultivars, finding that the starches showed A-type crystallinities, and had thermal and pasting properties that varied greatly among different ginkgo cultivars, suggesting a variation in cooked kernel quality among different cultivars [20]. Furthermore, the amylose content, amylose/amylopectin ratio, and crystalline structure and fine structure of amylose and amylopectin might greatly influence the physicochemical properties of ginkgo starches, including gelatinization, retrogradation, and pasting properties [20]. However, the structure of individual starch granules has yet to be determined, with the effects of starch granule structure and protein on the physicochemical properties of ginkgo kernel flours remaining unclear. Studies on rice flours have shown that the amylose content is among the most important factors affecting the physicochemical properties of flours, demonstrating that flours with different amylose contents exhibited distinct pasting properties [21]. The endogenous proteins and amylose activity were found to be largely responsible for observed variations in the pasting properties of rice starches and flours [22]. Furthermore, ectogenous proteins can decrease the pasting viscosity and increase the endotherm temperatures and gel behavior [23]. These studies on rice have implied that the amylose and protein structure play an important role in determining the flour physicochemical properties. Whether these findings for rice are applicable to ginkgo seed kernel flour has yet to be determined.

The grain quality of ginkgo largely depends on the physicochemical properties of kernel flours. Seven ginkgo cultivars commonly found in South China were used in this study. Their seed kernel morphology and components, together with starch granule fine structure, thermal and pasting properties of the corresponding kernel flours, and their interrelationships were investigated. Furthermore, in vitro experiments were conducted to analyze the effects of protein and starch structures on their physicochemical properties. This paper not only provides a comparative profiling of kernel starch and protein in terms of their structure and components, and their functional impact on the physicochemical properties of kernel flours, but also provides vital information for the potential improvement of ginkgo kernel quality for food and industrial applications.

## 2. Materials and Methods

### 2.1. Ginkgo Samples and Growth Conditions

Seven Ginkgo cultivars were selected in this study, namely *G. biloba* L. cv. ‘Qixingguo’ (QXG), *G. biloba* L. cv. ‘Dafozhi’ (DFZ), *G. biloba* L. cv. ‘Dongtinghuang’ (DTH), *G. biloba* L. cv. ‘Fozhi’ (FZ), *G. biloba* L. cv. ‘Maling’ (ML), *G. biloba* L. cv. ‘Dalongyan’ (DLY), and *G. biloba* L. cv. ‘Longyan’ (LY). Mature ginkgo seeds were collected from ten plants of the same variety at the Ginkgo experimental farm in Yangzhou University, in early October 2019.

The mature ginkgo seeds were harvested by a stripping machine (Xing Yuan-Xin/BGTP), and the nuts were dried in the shade for about a week. The ginkgo kernels were then shelled manually and dried in an oven, at 37 °C, for one week, prior to milling into flour, using a sample crusher (FOSS 1093 Cyclotec Sample Mill, Hoganas, Sweden).

### 2.2. Morphology and Weight of Ginkgo Kernels

The seed, nut, and kernel of ginkgo were photographed, using an EOS-60D Canon digital camera, as previously described [24]. The cellular distributions of starch and proteins in the kernel were observed by using scanning electron microscopy (SEM), as previously described [25]. The weight and size of ten ginkgo nuts or kernels, which were randomly selected, were measured by using an electronic balance and a Vernier caliper, respectively.

### 2.3. Major Component Analysis of Ginkgo Kernels

The moisture content (MC) was measured by using a halogen moisture analyzer (Mettler Toledo MJ33, Greifensee, Switzerland) [20]. The total starch content (TSC) of kernel flours was analyzed by using a total starch assay kit (K-TSTA; Megazyme, Wicklow, Ireland) [26]. The apparent amylose content (AAC) was measured by using an iodine colorimetric method with some modifications [18]. In detail, kernel flour (20 mg) was weighed accurately into a 10 mL screw-cap tube prior to adding DMSO (5 mL). The resulting suspension was thoroughly mixed and heated at 90 °C for 2 h, with intermittent vortexing. An aliquot of this test starch solution (500 μL) was pipetted into a 50 mL volumetric flask, followed by the addition of DMSO (500 μL) and iodine reagent (1 mL; 0.2% I_2_ and 2% KI, *w*/*v*). This mixture was diluted to 50 mL with distilled water, and the absorbance was measured at 620 nm. The amylose content was determined from a standard curve developed by using amylose and amylopectin blends. The crude protein content (CPC) was measured by using a nitrogen analyzer (Tecator Kjeltec 2300, FOSS, Hillerød, Denmark) according to AOAC standard method 990.03 [27]. The total amino acid content (TAAC) was analyzed by using a Hitachi L8900 Amino Acid Analyzer (Hitachi, Tokyo, Japan), following the method described by Yang et al., but with some modifications [28]. Briefly, dry kernel flour (10 mg) was weighed accurately into a 2 mL screw-cap tube prior to adding 6 N HCl (1 mL; Sigma, St. Louis, MO, USA) and L(+)-norleucine (10 nmol; Wako Pure Chemicals, Osaka, Japan). The samples were heated to 110 °C and incubated for 20 h prior to centrifugation at 6000× *g* for 10 min. Following removal of the supernatant, the pellet was dried by blowing with a nitrogen stream. The dried samples were then dissolved in Na-S buffer (1 mL) for 30 min in a mixer and extracted for 10 min by ultrasonication. After centrifugation (12,000× *g*, 10 min), the supernatant was collected and filtered through a 0.45 μm nylon membrane syringe filter (Pall Life Sciences, Ann Arbor, MI, USA) before instrumental analysis. The relative amino acid content was calculated and normalized with the level of L(+)-norleucine. For each measurement, two technical replicates were performed.

### 2.4. Isolation of Ginkgo Starch

Ginkgo starch was isolated by using the protease method, as described by Lu with some minor modifications [20]. Briefly, a sample of endosperm (10 g) was soaked in NaOH solution (pH 8.0), overnight, and then ground in a Waring blender (IKA-T RCT-Basic, Staufen, Germany) for 3 min. Following the addition of alkaline protease (50 mg/g) and sodium azide solution (25 μL, 40 mg/mL), the samples were incubated at 42 °C, with shaking (200 rpm) for 16 h. The resulting slurry was then passed through a 75 μm screen. After removing the soft top layer of the slurry, the sample containing starch was centrifuged at 3600× *g* for 10 min. The sediment was then washed with distilled water and centrifuged for 10 min, which was repeated five times. For lipid removal, the starch sediment was further washed with ethanol (100%) twice prior to adding CHCl_3_/methanol (30 mL; 1:1, *v*/*v*). Following incubation at 45 °C in a shaker with speed of 200 rpm for 2 h, the resulting starch slurry was centrifuged at 3600× *g* for 10 min. The starch precipitate was washed four times with 85% ethanol prior to drying in a convection oven at 40 °C for 48 h.

### 2.5. Morphology Analysis of Ginkgo Starch

A diluted sample of the starch slurry obtained above (1%, *w*/*v*) was mounted on a microscope slide in 50% glycerol aqueous solution. The morphology and Maltese cross pattern of starch were observed by confocal laser scanning microscopy (CLSM; Carl Zeiss, LSM880, Jena, Germany) under bright field and polarizing light models, respectively. Starch granules were stained with 8-aminopyrene-1,3,6-trisulfonic acid trisodium salt (APTS) (Beckman Coulter) prior to suspension in 50% glycerol (0.5 mL) and analyzed by using CLSM, as described by Naguleswaran [29].

### 2.6. Raman Spectroscopy Analysis of Ginkgo Starch

Raman images were acquired on a Thermo Scientific DXR xi Raman imaging microscope (Thermo Fisher Scientific, Madison, WI, USA). Raman spectra were collected over a 30 × 30 μm^2^ area, using a 50 × /0.7 Zeiss objective and a laser (10 mW, 532 nm) with a step size of 1.0 μm and an exposure time of 0.01 s per pixel, performing 10 scans per image, ranging from 200 to 2000 cm^−1^. Several sections of starch from seven ginkgo cultivars were examined, and images were recorded from different areas in each section. Raman images were analyzed by using Thermo Scientific OMNIC xi Raman imaging software (Thermo Fisher Scientific, Waltham, MA, USA).

### 2.7. Analysis of Ginkgo Flour Thermal and Pasting Properties

Thermal properties of both the native and gelatinized kernel flours were investigated by using differential scanning calorimetry (DSC) (Model 200 F3 Maia, NETZSCH, Weimar, Germany) [20]. Briefly, ginkgo kernel flour (5 mg) was mixed with deionized water (10 μL), placed in a sealed aluminum pan, and incubated at 4 °C, overnight. The samples were equilibrated at room temperature for 2 h before measurement. An empty pan was used as a reference to calibrate the DSC analyzer before placing the sample and heating from 15 to 120 °C, at a rate of 10 °C/min. In terms of the retrogradation measurement, the DSC pans containing the gelatinized samples (after gelatinization analysis) were stored at 4 °C for a week and prior to scanning. The DSC parameters included onset temperature (*T*_o_), peak temperature (*T*_p_), conclusion temperature (*T*_c_), and enthalpy of gelatinization (Δ*H*), which were set as default. Enthalpies were calculated on the kernel flour dry-weight basis. The pasting properties of kernel flours were analyzed by using a Rapid Visco Analyzer (RVA) (RVA-3D, Newport Scientific, Narrabeen, NSW, Australia) [30]. Briefly, ginkgo kernel flour (3 g) was placed in an aluminum canister containing 25 mL of distilled water, silver nitrate solution (AgNO_3_; 0.5 mmol/L), or dithiothreitol solution (DTT; 5 mmol/L). The viscosity was determined by using a standard procedure, as described by Zhang et al. [21].

### 2.8. Statistical Analysis

All data are presented as means ± standard deviation of three replicates. SPSS 25.0 statistical software was used to compare the data from one-way analysis of variance (ANOVA), Tukey’s test (*p* < 0.05), and the bivariate Pearson correlation. Principle component analysis (PCA) was performed by using the R programming language to further analyze the effects of starch and protein structure on the physicochemical properties of ginkgo kernel flour.

## 3. Results and Discussion

### 3.1. Morphology of Ginkgo Kernels

**Figure 1:**
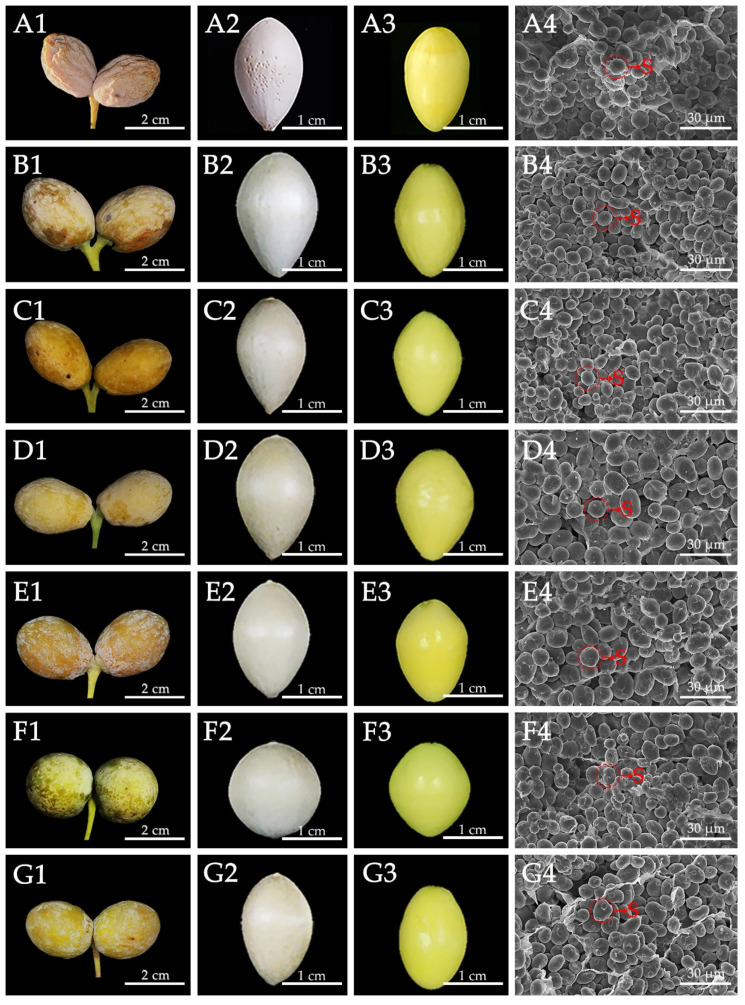
Morphologies of ginkgo seeds, nuts, kernels, and scanning electron micrographs (SEM) of kernel cross-section from different cultivars. (**A1**,**B1**,**C1**,**D1**,**G1**) Morphologies of ginkgo seeds; (**A2**,**B2**,**C2**,**D2**,**G2**) Morphologies of ginkgo nuts; (**A3**,**B3**,**C3**,**D3**,**G3**) Morphologies of ginkgo kernels; (**A4**,**B4**,**C4**,**D4**,**G4**) SEM of kernel cross-section; (**A**–**G**) Ginkgo cultivars Qixingguo (QXG), Dafozhi (DFZ), Dongtinghuang (DTH), Fozhi (FZ), Maling (ML), Dalongyan (DLY), and Longyan (LY); S, Starch; 1, Bars = 2 cm; 2, 3, Bars = 1 cm; 4, Bars = 30 μm.

The morphologies of matured seeds, nuts, and kernels from the seven ginkgo cultivars are shown in Figure 1. The seeds, nuts, and kernels were yellow (Figure 1(A1–G1)), white (Figure 1(A2–G2)), and chartreuse (Figure 1(A3–G3)) in color, respectively. The nuts were fusiform, and the kernels were oval in shape (Figure 1(A2–E2,G2,A3–E3,G3)), except for DLY, which had spherical nuts and kernels (Figure 1(F2,F3)). The surface of the QXG nut had numerous scrobiculi (Figure 1(A2)), while other nuts were smooth (Figure 1(B2–G2)). Significant differences in kernel weight were observed among the seven ginkgo cultivars, ranging from 0.86 (LY) to 1.72 g (FZ) (Table 1). The kernel size also varied significantly among the seven ginkgo cultivars (Table 1), ranging from 13.53 (LY) to 17.84 mm (DLY) in width, and from 20.85 (DLY) to 26.44 (DFZ) mm in length. These values were slightly higher than those reported for ginkgo kernels by Ch’ng [31]. DFZ had the largest kernels (26.44 × 16.04 mm), followed by QXG (26.21 × 15.24 mm) and FZ (25.67 × 15.20 mm), while LY had the smallest kernels (22.38 × 13.53 mm). Consistent with our previous report [20], SEM studies showed no discernible variation in the external morphology of the starch granules among the seven ginkgo kernels (Figure 1(A4–G4)). The above results suggested extensive morphology variation among the ginkgo seeds from different cultivars, which was consistent with our previous study [32].

### 3.2. Major Components of Ginkgo Kernel

The MC, TSC, AAC, CPC, and TAAC values of the seven ginkgo kernels were compared, as shown in Table 1. The MCs of the seven ginkgo dry kernels flours were similar, ranging from 6.40% (DLY) to 7.42% (ML), which were lower than those of the fresh kernel (41%) and ginkgo starch (10.65–11.30%) [7,20,31]. The TSC showed significant differences among the seven cultivars, ranging from 68.95% (DLY) to 71.83% (ML), consistent with previous reports (60–70%) [4,12]. The AACs showed significant variation among the seven ginkgo kernels, ranging from 18.58% (LY) to 25.61% (ML), which might be attributed to distinct genotypic differences among the seven ginkgo cultivars [33].

The nutritional value of ginkgo kernel is commonly measured by its protein content and amino acid composition, which are also the key parameters used for determining ginkgo grain quality [12,14]. The desire for the improvements in nutritional value has prompted the investigation in the protein content and amino acid profiles of ginkgo kernels. As shown in Table 1, CPCs and TAACs of the kernels significantly differed among the seven ginkgo cultivars, with ranges of 6.88–12.57% and 6.82–9.30%, respectively. For DTH, ML, and QXG, the CPC values were 10.39%, 10.20%, and 9.94%, respectively, which were lower than those of DFZ and DLY, but higher than those of LY and FZ. A total of 15 amino acids were identified in the ginkgo kernels in all the seven cultivars with slight variations among them (Supplementary Materials Table S1). The two sulfur-rich amino acids, cysteine (Cys) and methionine (Met), were not discernible in the ginkgo kernels, probably owing to their very low contents and possible losses during acid hydrolysis [34]. Among the 15 amino acids detected, glutamic acid (Glu) (1.31–1.56) and arginine (Arg) (0.68–1.08) had among the highest contents, while histidine (His) (0.14–0.20) had the lowest content, which agreed with a previous report by Zhou et al. [14]. Glu is the most abundant amino acid and a major neurotransmitter in the brain, which is crucial for brain cellular metabolism [35]. Arg plays a central role in several biological pathways, such as cell growth, proliferation, and survival [36]. The high levels of Glu and Arg reported in this study are a key feature of ginkgo four, which might have potential beneficial effects on human health.

### 3.3. Starch Granule Structures in Ginkgo Kernel

**Figure 2:**
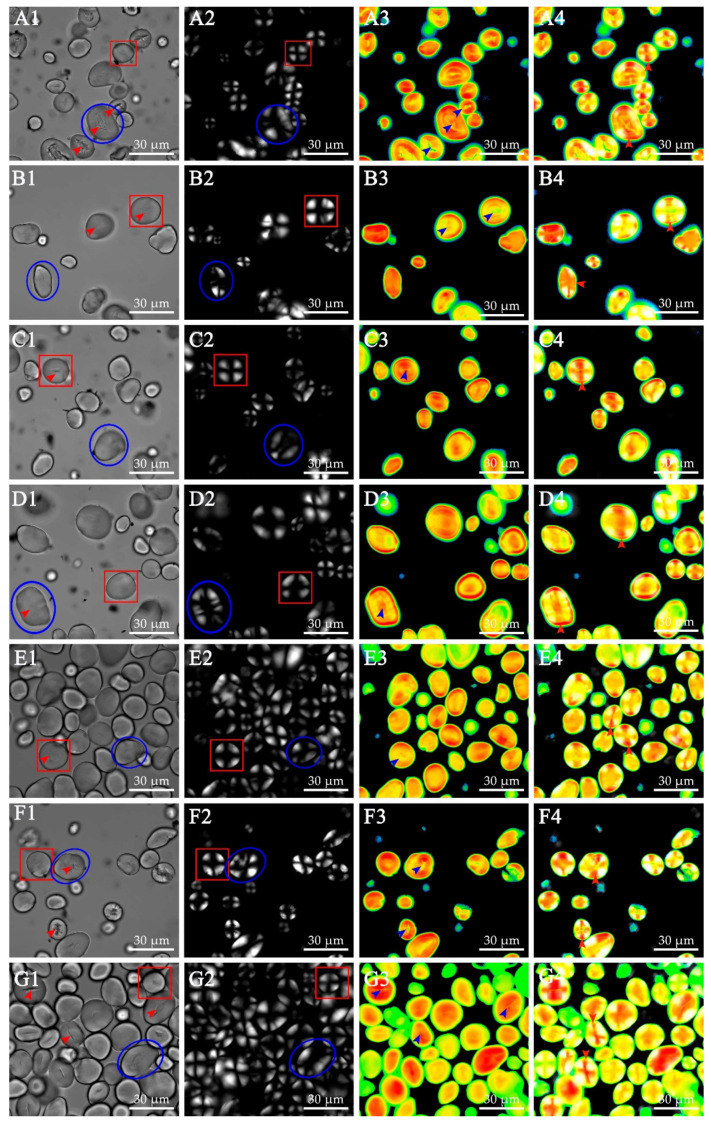
Photomicrographs of ginkgo starches from different cultivars. (**A1**–**G1**) Normal light microscopy (NLM); (**A2**–**G2**) Polarized light microscopy (PLM); (**A3**–**G3**) Confocal laser scanning microscopy (CLSM); (**A4**–**G4**) Merged images of PLM and CLSM; (**A**–**G**) Ginkgo cultivars QXG, DFZ, DTH, FZ, ML, DLY, and LY. Bars = 30 μm.

The microstructure of ginkgo starch was observed by using CLSM under bright field, polarizing light, and laser light models (Figure 2). For each cultivar, the ginkgo starch exhibited significantly heterogeneous shapes, including spherical (Figure 2(A1–G1), red square), ellipsoid, or irregular shapes (Figure 2(A1–G1), blue circle). The ellipsoid and irregular starch granules were larger than the spherical granules (Figure 2(A1–G1)). Furthermore, the spherical starch granules showed regular Maltese cross patterns (Figure 2(A2–G2), red square), while the ellipsoid or irregular-shaped starch granules showed irregular Maltese cross patterns in the polarizing microscope image (Figure 2(A2–G2), blue circle). Normally, the intact shapes of starch granules were birefringent and showed characteristic Maltese cross patterns under polarizing light. The birefringence patterns indicated the radial alignment of crystallites within granules [37], suggesting that different Maltese cross patterns were due to differences in the radial alignment of crystallites. Therefore, variations in the radial alignment of crystallites were observed for different starch shapes. As shown in Supplementary Materials Table S2, the percentage of regular and irregular Maltese cross patterns varied among the seven ginkgo starches in the ranges of 41.28–58.93% and 41.07–58.72%, respectively. Consequently, significant variations were observed among the seven cultivars.

Starch consists of amylose and amylopectin, with the amylose content and distribution playing important roles in the study of starch quality and function. Staining starch granules with APTS enables visualization of the distribution of amylose and amylopectin in starch granules by CLSM. APTS reacts specifically with the reducing end of starch molecules, leading to a 1:1 stoichiometric ratio of starch molecule labeling. Amylose is labeled with more APTS, owing to its higher molar ratio of reducing ends compared with amylopectin, resulting in a positive correlation between fluorescence intensity and amylose content, and enabling the distinction of amylose and amylopectin [38]. In this study, CLSM images of ginkgo starch granules showed clear amorphous growth rings around a central hilum. According to the analysis software, colors representing the fluorescence intensity (i.e., amylose content) from low to high were black, blue, green, yellow, and red. As shown in Figure 2(A3–G3), clear intense fluorescent bands were found for most of the starch granules, indicating that amylose was mainly distributed in amorphous growth rings. It is of interest to note that a cave was observed in the middle of a starch granule, as observed in the bright light images (Figure 2(A1–G1), red arrow). The cave was positioned to the area with low fluorescence within a starch granule (Figure 2(A3–G3), blue arrow), indicating a low level of amylose distribution in this region. Recent studies reported that some cereal species, such as rice, tend to have caves in the central region of a single starch granule when its amylose content is low [39]. In the CLSM image, a weak fluorescent dot in the central region of a starch granule was barely discernible in the kernels of ML cultivar, which might be attributable to their high AAC levels (Figure 2(E1–E4) and Table 1). Further, as shown in the merged images of PLM and CLSM, the intense fluorescent dots were well overlapped with the Maltese cross, indicating the high amylose concentration in this region. However, this is inconsistent with a study in corn starch granules where a strong fluorescent dot was observed in the central region [40]. Such a discrepancy suggests that the internal structure of ginkgo starch might be different from that of a typical cereal starch, which should be taken into account in future structural analysis of starch granules.

**Figure 3:**
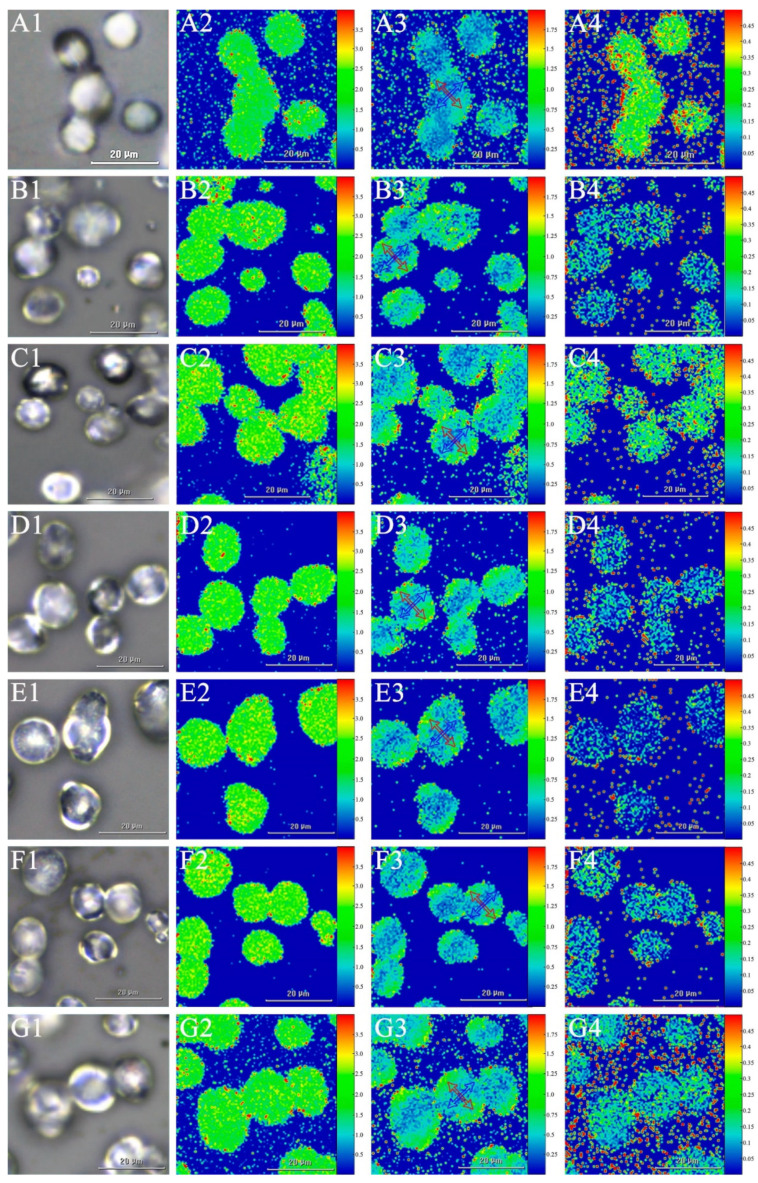
Laser Raman maps of ginkgo starches from different cultivars. (**A1**–**G1**) Light micrographs of unstained ginkgo starches; (**A2**–**G2**) Map of 480/942 cm^−1^ band ratio, indicating crystallinity; (**A3**–**G3**) Map of 865/942 cm^−1^ band ratio, indicating chain orientation; (**A4**–**G4**) Map of 952/942 cm^−1^ band ratio, indicating starch branching. (**A**–**G**) Ginkgo cultivars QXG, DFZ, DTH, FZ, ML, DLY, and LY. Bars = 20 μm.

The internal structures of starch granules were further investigated, using Raman imaging microscopy, which can micromap the composition and physical state of individual starch granules [37]. Unstained white-light images and Raman images of ginkgo starches, calculated from the intensities measured in the region of 2000–200 cm^−1^, are shown in Figure 3. The 480/942 cm^−1^ ratio was used as a standard for crystallinity [41], with the plotted crystallinity maps for starches derived from the seven ginkgo cultivars shown in Figure 3(A2–G2). This map was homogeneous within an individual starch granule and between starch granules of a single cultivar. However, the 480/942 cm^−1^ ratio showed differences among ginkgo starches from the seven cultivars, ranging from 1.89 ± 0.01 to 2.09 ± 0.02 (Supplementary Materials Table S2). This indicated variations in the crystallinity among the seven ginkgo starches, in agreement with our previous study [20]. The 865/942 cm^−1^ ratio was used as an indicator of the radial orientation of crystallites (chain orientation) within the individual starch granules [33,42,43]. As shown in Figure 3(A3–G3), the map showed a spatial variation within the individual starch granules, with lower values in the horizontal direction (Figure 3(A3–G3), blue arrow) relative to the vertical direction (Figure 3(A3–G3), red arrow), which was congruent with the above results from CLSM (Figure 2(A3–G3)). Furthermore, the 865/942 cm^−1^ ratio, in both the vertical and horizontal direction regions, varied among the ginkgo starches derived from different cultivars, ranging from 0.69 ± 0.05 to 0.96 ± 0.07, and from 0.33 ± 0.02 to 0.53 ± 0.03, respectively (Supplementary Materials Table S2). Considering the findings in cereals [29,44], we hypothesized that horizontal areas in a starch granule that showed lower orientation might be correlated with the amylose content. In fact, as shown in Figure 2(A4–G4), a cross structure was observed in almost every starch granule, indicating a high amylose concentration in the non-birefringent area, which further supported this hypothesis. The 952/942 cm^−1^ ratio was used to determine the ratio of linear residues to branched residues (branching) within starch granules [33]. Therefore, a higher 952/942 cm^−1^ ratio corresponded to a higher linear-to-branched ratio. The ratio in the map was homogeneous for ginkgo starches, both within individual and between starch granules within a cultivar (Figure 3(A4–G4)). However, the 952/942 cm^−1^ ratio varied among starches from the seven ginkgo cultivars, ranging from 0.11 to 0.22 (Supplementary Materials Table S2). This indicated variation in starch branching among the seven cultivars, which agreed with our previous starch molecular weight distribution analysis [20].

### 3.4. Thermal Properties of Kernel Flours

The gelatinization properties of the seven ginkgo kernel flours were determined by DSC. The seven ginkgo kernel flours showed similarly broad endothermic peaks (Supplementary Materials Figure S1A). The gelatinization temperatures and ΔH_gel_ values varied slightly among the seven ginkgo kernel flours (Table 2). The *T*_o_, *T*_p_, and *T*_c_ values for the kernel flours were in the ranges of 74.90–79.30 °C, 81.50–82.65 °C, and 89.05–91.70 °C, respectively, and were higher than the corresponding values of ginkgo starch (67.23–71.21 °C, 73.68–76.75 °C, and 76.17–84.19 °C, respectively) that were reported in our previous study [20]. The ΔH values ranged from 9.15 to 11.76 J·G^−1^, which were lower than the corresponding values of ginkgo starch (9.34–12.86 J·G^−1^) in our previous report [20]. It suffices to note that the gelatinization temperatures of ginkgo kernel flours are higher than those of normal cereal starch [21], which could be the unique features of ginkgo starch. Studies from other starch systems have also suggested that the gelatinization properties are largely determined by the chemical composition, such as the contents of lipids, proteins, starch, and dietary fiber [45]. It is plausible to hypothesize that the relatively high gelatinization temperatures of ginkgo kernel flours are attributable to its peculiar chemical compositions.

Furthermore, the retrogradation properties of the gelatinized flours were further analyzed for all seven ginkgo kernel flours, showing similar retrogradation curves (Supplementary Materials Figure S1B). The *T*_o_’, *T*_p_’, and T_c_’ retrogradation temperatures and retrogradation enthalpy (Δ*H_ret_*) varied among the seven ginkgo kernel flours, giving ranges of 44.00–46.70 °C, 54.80–58.80 °C, 66.90–68.45 °C, and 3.11–4.24 J·G^−1^, respectively (Table 2). During retrogradation, amylose forms double-helical associations, while amylopectin crystallization occurs through association of the outermost short branches [46]. The high *T_p_*’ for cultivar ML might therefore be attributable to its high AAC value. In addition to the amylose content, the fine molecular structure of starch granule also plays a role in determining the ordering/amount of amylopectin double helices and hence the starch gelatinization profiles [43]. The cultivar ML showed the highest 865/942 band ratio in the analysis of Raman spectra parameters, which are consistent with its observed relatively high *T*_p_’ and *T*_c_’ values. It, however, merits further investigation in the relationships between gelatinization profiles and starch fine structures.

**Figure 4:**
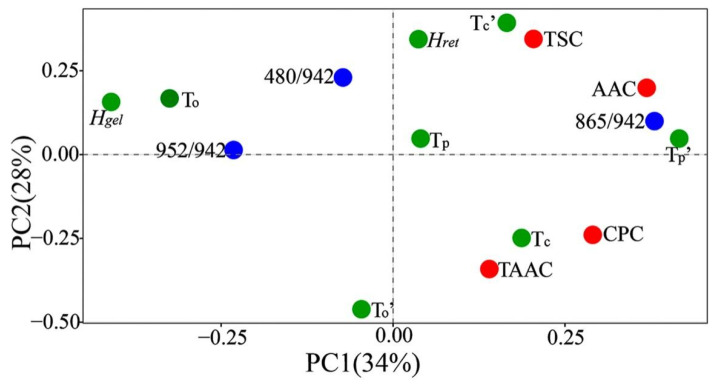
PCA loading plots of basic compositions, Raman spectra ratios, and thermal parameters of kernel flours from seven ginkgo cultivars. Red, blue, and green represent basic compositions, Raman spectra ratios, and thermal properties, respectively.

Pearson correlation coefficients were calculated to analyze the factors affecting the thermal properties of ginkgo kernel flours (Supplementary Materials Table S3). The TSC mainly positively affected *T*_p_’ (*r* = 0.87, *p* < 0.05). The AAC positively affected *T*_p_’ (*r* = 0.82, *p* < 0.05) and *T*_c_’ (*r* = 0.72, *p* < 0.05). The CPC was significantly negatively correlated with Δ*H_gel_* (*r* = −0.70, *p* < 0.05). The 865/942 ratio was significantly positively correlated with *T*_c_’, but negatively correlated with Δ*H_gel_* and *T*_o_’. The 952/942 ratio was negatively correlated with *T*_c_’, but positively correlated with Δ*H_gel_* and *T*_o_’. PCA was performed to further analyze the relationships between the basic components and thermal properties of kernel flours from the seven ginkgo cultivars (Figure 4). PC1 and PC2 described 34% and 28% of the total variance, respectively. TSC and *T*_p_’, AAC and *T*_p_’, AAC and *T*_c_’, the 865/942 ratio and *T*_c_’, the 952/942 ratio and Δ*H_gel_*, and the 952/942 ratio and *T*_o_’ were close to each other on the loading plot, indicating that they had positive correlations, while CPC and Δ*H_gel_*, the 865/942 ratio and Δ*H_gel_*, the 865/942 ratio and *T*_o_’, and the 952/942 ratio and *T*_c_’ were all negatively correlated. These results indicated that starch composition, TSC, and CPC played important roles in determining the retrogradation properties of ginkgo kernel flours. Furthermore, the Raman parameters reflected the proportion of crystallites associated with the molecular order, which plays an important role in determining starch thermal properties in ginkgo, in a similar fashion to cereal starch [18].

### 3.5. Pasting Properties of Kernel Flours

**Figure 5:**
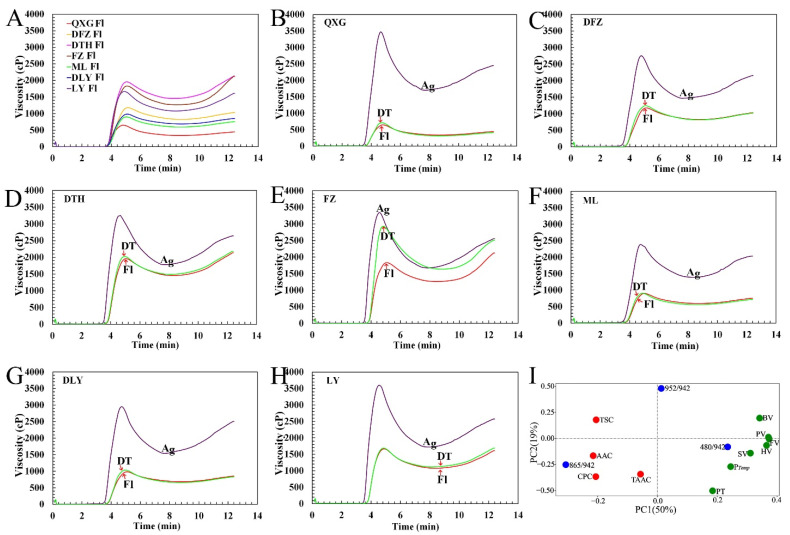
(**A**–**H**) Rapid viscosity profiles with different treatments. (**I**) PCA loading plots of basic compositions, Raman spectra ratios, and pasting parameters of kernel flours from seven ginkgo cultivars. Rapid viscosity profiles of (**A**) kernel flours from seven ginkgo cultivars in water, and (**B**–**H**) different kernel flours in water, AgNO_3_, and DTT ((**B**) QXG, (**C**) DFZ, (**D**) DTH, (**E**) FZ, (**F**) ML, (**G**) DLY, and (**H**) LY). Red, blue, and green represent basic compositions, Raman spectra ratios, and physicochemical properties, respectively.

The pasting properties of kernel flours from seven ginkgo cultivars that were measured by using the RVA are summarized in Figure 5A. The seven ginkgo kernel flours showed significantly different pasting properties at 100 g/kg solids. Compared with ginkgo starch [20], the kernel flours showed a lower peak viscosity (PV) (532–1946.5 cP), breakdown viscosity (BV) (110.0–581.5 cP), hot viscosity (HV) (327.5–1446.0 cP), final viscosity (FV) (427.0–2121.0 cP), and setback viscosity (SV) (−192.3 to 204.7 cP) (Supplementary Materials Table S4). These results indicated that some endogenous factors other than starch had affected the pasting properties of ginkgo kernel flours. This is in agreement with some studies on cereal grain flours that, in addition to starch molecular structure, α-amylase and starch-granule-associated proteins also play important roles in affecting the pasting properties of starch [47,48].

The Pearson correlation and PCA were used to analyze the relationship between RVA characteristics and other physicochemical properties. As shown in Supplementary Materials Table S3, the BV was significantly negatively correlated with AAC (*r* = −0.77, *p* < 0.05) and the 865/942 cm^−1^ ratio (r = −0.77, *p* < 0.05), while the PV (r = 0.70, *p* < 0.05), HV (r = 0.75, *p* < 0.05), FV (r = 0.72, *p* < 0.05), and SV (r = 0.76, *p* < 0.05) were positively correlated with the 480/942 cm^−1^ ratio, respectively. A negative correlation was observed between the 952/942 cm^−1^ ratio and P*_Temp_* (r = −0.66, *p* < 0.05). PCA was also performed, with PC1 and PC2 describing 50% and 19% of the variance, respectively (Figure 5I). Similarly, the PCA loading plot indicated that BV was located opposite AAC and the 865/942 cm^−1^ ratio, and that P*_Temp_* was located opposite the 952/942 cm^−1^ ratio, indicating that the BV was negatively correlated with AAC and the 865/942 cm^−1^ ratio, while P*_Temp_* was negatively correlated with the 952/942 cm^−1^ ratio. This was consistent with our previous studies on the influence of AAC on BV [20]. The 480/942 cm^−1^ ratio was close to PV, HV, FV, and SV in the PCA loading plot, indicating that these parameters were positively correlated. These results indicated that the pasting properties of ginkgo kernel flours were affected by the kernel AAC and starch fine structure. These results were congruent with previous reports on some other cereals, such as rice and maize [49].

### 3.6. Effects of α-Amylase and Protein Structure on Pasting Properties of Kernel Flours

Although starch is the most abundant ingredient in ginkgo kernel flour, its pasting properties differed from those of the ginkgo flours [20]. The α-amylase and starch-granule-associated proteins are known to be important factors affecting the RVA pasting properties in rice [50]. In this study, to examine the effect of α-amylase on the pasting properties of ginkgo kernel flours, the endogenous α-amylase activity was reduced by adding AgNO_3_ solution (0.5 mmol/L), which is an α-amylase inhibitor, to the kernel flours, in comparison with adding water as a control. When AgNO_3_ solution was added to the ginkgo kernel flours, significant increases in the PV, HV, BV, and FV were observed, accompanied by large decreases in the SV, pasting time, and pasting temperature (Figure 5B–H and Supplementary Materials Table S4). Moreover, α-Amylase has been established to randomly hydrolyze the interior α-1,4-glucosidic linkage of starch, with even a few internal cleavages of amylopectin leading to a reduction in the molecular weight and, therefore, lower viscosity of the starch paste [22]. Therefore, endogenous α-amylase in ginkgo kernel flour is an important factor affecting paste viscosity. Similar results have been reported for some cereals, such as rice and wheat [22,51].

Protein content is an important factor influencing the palatability of some cooked cereals, such as cooked rice grain [52]. To examine the effect of protein structure on the pasting properties of ginkgo kernel flours, protein disulfide bonds were disrupted by adding DTT solution (5 mmol/L) to the kernel flours. Except for FZ, other six ginkgo cultivars all showed a slightly increased pasting curve in DTT solution relative to those in aqueous solution (Figure 5B–H and Supplementary Materials Table S4). However, the effect of protein structure on pasting profiles was rather minor compared with the AgNO_3_ treatment. The cultivar FZ, which has atypical RVA profiles, is a good sample for future study. The fact that the two sulfur-rich amino acids, Cys and Met, were not discernible in the ginkgo kernels proteins, as shown in Supplementary Materials Table S1, may suggest that less intermolecular and/or intramolecular disulfide bridges may have formed and, hence, have little effect on RVA profiles. Instead, the pasting properties of ginkgo flours were influenced greatly by α-amylase.

## 4. Conclusions

Ginkgo kernels are the main edible part of the seed, and recent studies have so far mainly focused on the structures and physicochemical properties of ginkgo starches, but the physicochemical properties of ginkgo kernel flours were less understood. In this study, we investigated the chemical components in flour, structural variations in starch granules, and their relationships with the physicochemical properties of kernel flours. Considerable variations in the chemical composition and physicochemical properties were observed among the seven ginkgo kernels’ flour samples. The novelty of this study comprises the new findings in the fine structure of starch granule, and the intricate relationships between pasting, thermal properties, and chemical components in ginkgo kernels four. The influence of starch and non-starch components varied on the different aspects of physicochemical properties of the ginkgo kernels flour, elucidating kernel flour as a complex system with multiple components. Correlation analysis showed that the properties of ginkgo flour (especially the pasting properties) were, to a large extent, determined by α-amylase activity. Furthermore, with the aid of chemical analysis, it was concluded that the gelatinization properties and retrogradation properties of kernel flours were affected by CPC, TAC, AAC, starch crystallinity, starch chain orientation, and starch branching. This study may help elucidate the relationships between the starch structure and physicochemical properties of ginkgo kernel flours, and provide vital information for ginkgo kernel applications in both food and non-food industries.

## Figures and Tables

**Table 1:** Morphologies and basic compositions of kernels from seven ginkgo cultivars.

Measured Parameters	Ginkgo Cultivars						
	QXG	DFZ	DTH	FZ	ML	DLY	LY
Shape	Oval	Oval	Oval	Oval	Oval	Spherical	Oval
Width (mm)	15.24 ± 0.36 ^b^	16.04 ± 0.39 ^b^	14.01 ± 0.29 ^c^	15.20 ± 0.27 ^b^	15.17 ± 0.48 ^b^	17.84 ± 0.72 ^a^	13.53 ± 0.17 ^c^
Length (mm)	26.21 ± 0.45 ^a^	26.44 ± 0.34 ^a^	23.63 ± 0.20 ^b^	25.67 ± 0.67 ^a^	24.50 ± 0.39 ^b^	20.85 ± 0.50 ^c^	22.38 ± 0.48 ^b^
Weight (g)	0.89 ± 0.11 ^d^	1.15 ± 0.30 ^bc^	1.03 ± 0.17 ^c^	1.72 ± 0.34 ^a^	1.35 ± 0.40 ^b^	1.16 ± 0.28 ^bc^	0.86 ± 0.07 ^d^
MC (%, *w*/*w*)	7.01 ± 0.26 ^a^	6.61 ± 0.05 ^a^	6.53 ± 0.18 ^a^	7.39 ± 0.76 ^a^	7.42 ± 0.26 ^a^	6.40 ± 0.12 ^a^	6.71 ± 0.11 ^a^
TSC (%, *w*/*w*)	70.72 ± 2.20 ^b^	70.45 ± 1.55 ^b^	70.24 ± 0.70 ^b^	69.40 ± 2.03 ^c^	71.83 ± 1.57 ^a^	68.95 ± 1.70 ^c^	70.45 ± 2.86 ^b^
AAC (%, *w*/*w*)	19.52 ± 1.03 ^bc^	20.26 ± 0.70 ^b^	20.25 ± 0.67 ^b^	18.96 ± 0.62 ^c^	25.61 ± 0.30 ^a^	19.44 ± 0.43 ^b^	18.58 ± 0.25 ^c^
CPC (%, *w*/*w*)	9.94 ± 0.03 ^b^	12.57 ± 0.04 ^a^	10.39 ± 0.01 ^b^	6.88 ± 0.02 ^d^	10.20 ± 0.13 ^b^	12.03 ± 0.01 ^a^	8.25 ± 0.03 ^c^
TAAC (mg/g)	7.43 ± 0.33 ^c^	7.94 ± 3.31 ^c^	8.80 ± 1.03 ^b^	6.82 ± 0.52 ^c^	7.77 ± 0.13 ^c^	9.30 ± 0.47 ^a^	7.47 ± 0.40 ^c^

Data are given as means ± standard deviation (*n* = 3). Values with the same letter in a row of the same cultivar are not significantly different (*p* < 0.05). MC, TSC, AAC, CPC, and TAAC are the moisture content, total starch content, apparent amylose content, crude protein content, and total amino acid content of ginkgo kernel flours, respectively.

**Table 2:** Thermal properties of seven ginkgo powders, as determined by differential scanning calorimetry.

DSC	Cultivars	*T*_o_ (°C)	*T*_p_ (°C)	*T*_c_ (°C)	Δ*H* (J/g)
Gelatinization	QXG	75.85 ± 0.24 ^bc^	81.90 ± 0.21 ^ab^	91.70 ± 0.02 ^a^	10.39 ± 0.61 ^b^
	DFZ	76.55 ± 0.14 ^b^	82.60 ± 0.06 ^a^	91.55 ± 0.28 ^a^	9.42 ± 0.15 ^c^
	DTH	75.70 ± 0.09 ^bc^	81.50 ± 0.15 ^ab^	89.95 ± 0.28 ^b^	10.43 ± 0.01 ^b^
	FZ	76.70 ± 0.05 ^b^	81.95 ± 0.03 ^ab^	89.95 ± 0.12 ^b^	10.54 ± 0.15 ^b^
	ML	74.90 ± 0.28 ^bc^	82.10 ± 0.28 ^a^	90.05 ± 0.35 ^b^	9.15 ± 0.46 ^c^
	DLY	75.95 ± 0.09 ^bc^	82.55 ± 0.08 ^a^	90.85 ± 0.27 ^a^	9.33 ± 0.04 ^c^
	LY	79.30 ± 1.75 ^a^	82.65 ± 0.78 ^a^	89.05 ± 0.16 ^b^	11.76 ± 0.71 ^a^
Retrogradation	QXG	45.80 ± 0.04 ^a^	55.80 ± 0.04 ^b^	66.90 ± 0.04 ^b^	4.24 ± 0.05 ^a^
	DFZ	44.40 ± 1.10 ^b^	56.95 ± 0.15 ^ab^	67.60 ± 0.10 ^ab^	4.04 ± 0.51 ^a^
	DTH	45.80 ± 0.05 ^a^	56.50 ± 0.11 ^b^	67.00 ± 0.07 ^b^	3.54 ± 0.23 ^ab^
	FZ	45.30 ± 0.20 ^ab^	56.10 ± 0.71 ^b^	67.45 ± 0.34 ^ab^	3.97 ± 0.16 ^a^
	ML	44.15 ± 0.49 ^b^	58.80 ± 1.98 ^a^	68.45 ± 0.92 ^a^	4.12 ± 0.23 ^a^
	DLY	46.70 ± 0.04 ^a^	56.70 ± 0.03 ^b^	67.20 ± 0.02 ^ab^	3.11 ± 0.04 ^b^
	LY	44.00 ± 0.02 ^b^	54.80 ± 0.04 ^b^	68.00 ± 0.08 ^a^	3.86 ± 0.03 ^ab^

Data are given as means ± standard deviation (*n* = 3). Values in the same column with different letters are significantly different (*p* < 0.05). *T*_o_, *T*_p_, *T*_c_, and Δ*H* correspond to the onset temperature, peak temperature, conclusion temperature, and enthalpy of gelatinization (*T*_o_, *T*_p_, *T*_c_, and Δ*H_gel_*) and retrogradation (*T*_o_’, *T*_p_’, *T*_c_’, and Δ*H_ret_*), respectively.

## Data Availability

The data presented in this study are available on request from the corresponding author.

## Supplementary Materials

The following are available online at https://www.mdpi.com/article/10.3390/foods10081721/s1. Supplementary Figure S1: Gelatinization (A) and retrogradation (B) of kernel flours from seven ginkgo cultivars as determined by DSC. Supplementary Table S1: Amino acid contents of kernels from seven ginkgo cultivars. Supplementary Table S2: Maltese cross differences and Raman spectra ratios of ginkgo starches from seven cultivars. Supplementary Table S3: Pearson correlation coefficients for basic compositions, starch structure parameters, thermal properties, and pasting properties of kernel powders from seven ginkgo cultivars. Supplementary Table S4: Pasting properties of kernel powders from seven gingko cultivars treated with water, DDT, and AgNO_3_.
