# Supplementary material for: Comparative Study of Physicochemical Properties and Starch Granule Structure in Seven Ginkgo Kernel Flours
Source: Foods. 2021 Jul 26;10(8):1721. doi: 10.3390/foods10081721 (PMC8392216; doi:10.3390/foods10081721)
Supplement: Supplementary file 1 [file foods-10-01721-s001.zip › foods-1253265-SI.pdf]

Supplementary Figure

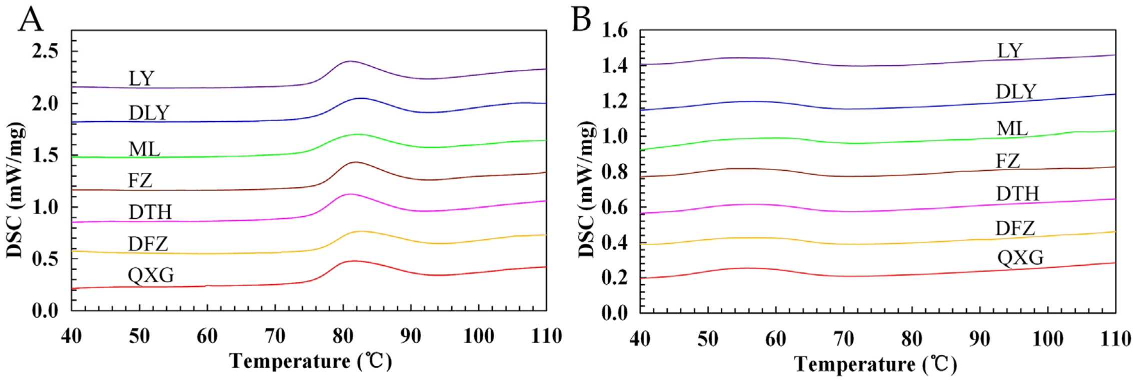

Figure S1. Gelatinisation (A) and retrogradation (B) of kernel flours from seven ginkgo cultivars as determined by DSC.

Supplementary Tables

Table S1. Amino acid contents of kernels from seven ginkgo cultivars

Items	Ginkgo cultivars						
	QXG	DFZ	DTH	FZ	ML	DLY	LY
Aspartic acid (Asp)	0.74±0.01[b]	0.81±0.32[a]	0.92±0.13[a]	0.71±0.10[b]	0.81±0.05[a]	0.94±0.04[a]	0.72±0.06[b]
Threonine (Thr)	0.41±0.00[a]	0.41±0.18[a]	0.45±0.06[a]	0.37±0.03[b]	0.40±0.00[a]	0.50±0.01[a]	0.39±0.02[b]
Serine (Ser)	0.49±0.00[b]	0.45±0.19[b]	0.54±0.07[a]	0.42±0.00[a]	0.47±0.00[b]	0.61±0.01[a]	0.48±0.02[b]
Glutamic acid (Glu)	1.31±0.01[b]	1.39±0.56[b]	1.56±0.16[a]	1.27±0.07[b]	1.34±0.01[b]	1.72±0.07[a]	1.31±0.06[b]
Glycin (Gly)	0.40±0.01[ab]	0.41±0.17[ab]	0.47±0.05[a]	0.36±0.03[b]	0.40±0.01[ab]	0.48±0.03[a]	0.39±0.03[ab]
Alanine (Ala)	0.51±0.01[b]	0.57±0.25[a]	0.63±0.08[a]	0.48±0.04[b]	0.54±0.02[b]	0.66±0.05[a]	0.52±0.05[b]
Valine (Val)	0.47±0.07[a]	0.53±0.20[a]	0.54±0.06[a]	0.42±0.03[b]	0.46±0.04[a]	0.54±0.06[a]	0.45±0.05[ab]
Isoleucine (Ile)	0.36±0.04[a]	0.43±0.19[a]	0.44±0.08[a]	0.33±0.02[a]	0.39±0.03[a]	0.47±0.06[a]	0.38±0.06[a]
Leucine (Leu)	0.56±0.03[ab]	0.62±0.25[a]	0.69±0.08[a]	0.54±0.04[b]	0.60±0.03[a]	0.71±0.05[a]	0.58±0.05[a]
Tyrosine (Tyr)	0.20±0.01[a]	0.21±0.10[a]	0.23±0.03[a]	0.18±0.02[a]	0.20±0.00[a]	0.25±0.00[a]	0.20±0.02[a]
Phenylalanine (Phe)	0.35±0.00[a]	0.36±0.17[a]	0.42±0.06[a]	0.36±0.02[a]	0.36±0.00[a]	0.42±0.00[a]	0.37±0.02[a]
Histidine (His)	0.16±0.01[a]	0.17±0.08[a]	0.19±0.03[a]	0.14±0.01[a]	0.18±0.00[a]	0.20±0.01[a]	0.16±0.02[a]
Lysine (Lys)	0.34±0.00[a]	0.35±0.15[a]	0.45±0.05[a]	0.35±0.03[a]	0.38±0.00[a]	0.42±0.02[a]	0.37±0.03[a]
Arginine (Arg)	0.91±0.16[a]	0.93±0.36[a]	0.92±0.08[a]	0.68±0.04[b]	0.91±0.01[a]	0.11±0.07[c]	0.91±0.11[a]
Proline (Pro)	0.23±0.01[b]	0.30±0.14[a]	0.32±0.02[a]	0.22±0.04[b]	0.36±0.02[a]	0.31±0.00[a]	0.25±0.02[b]

Data are given as means ± standard deviation (n=3). Values with the same letter in the same row are not significantly different ($p < 0.05$). Data are presented as mg/g dry kernel weight.

Supplementary Table S2. Maltese cross differences and Raman spectra ratios of ginkgo starches from seven cultivars

Samples	Regular Maltese crosses（%）	Irregular Maltese crosses（%）	480/942 cm^{-1}	865/942 cm^{-1}			952/942 cm^{-1}
				Periphery of starch	Centre of starch	Whole starch	
QXG	41.28±1.8^{cd}	58.72±1.81^{a}	1.89±0.01^{b}	0.69±0.05^{cd}	0.41±0.05^{b}	0.54±0.04^{b}	0.22±0.01^{a}
DFZ	58.93±2.93^{a}	41.07±2.93^{c}	2.03±0.02^{a}	0.87±0.06^{b}	0.40±0.02^{b}	0.65±0.10^{a}	0.11±0.00^{b}
DTH	54.07±1.82^{ab}	45.93±1.82^{bc}	2.09±0.02^{a}	0.77±0.10^{c}	0.33±0.02^{c}	0.48±0.01^{c}	0.21±0.01^{a}
FZ	43.91±3.05^{c}	56.09±3.05^{a}	2.01±0.01^{a}	0.61±0.02^{d}	0.33±0.03^{c}	0.44±0.03^{c}	0.10±0.01^{b}
ML	51.79±5.96^{ab}	48.21±5.96^{bc}	2.00±0.03^{a}	0.96±0.07^{a}	0.53±0.03^{a}	0.69±0.03^{a}	0.11±0.01^{b}
DLY	54.28±0.62^{ab}	45.72±0.62^{bc}	1.89±0.04^{b}	0.86±0.11^{b}	0.42±0.01^{b}	0.59±0.01^{b}	0.11±0.01^{b}
LY	48.54±4.21^{b}	51.46±4.21^{ab}	2.02±0.09^{a}	0.74±0.04^{c}	0.40±0.02^{b}	0.53±0.07^{b}	0.21±0.01^{a}

Data are given as means ± standard deviation (n = 3). Values with the same letter in a column of the same cultivar are not significantly different ($p < 0.05$).

Supplementary Table S3. Pearson correlation coefficients for basic compositions, starch structure parameters, thermal properties, and pasting properties of kernel powders from seven ginkgo cultivars.

Items	TSC	AAC	CPC	TAAC	480/942	865/942	952/942
T_o	-0.22	-0.62	-0.42	-0.33	0.20	-0.39	0.28
T_p	-0.14	-0.16	0.28	0.05	-0.26	0.39	-0.35
T_c	-0.03	-0.03	0.63	0.15	-0.55	0.16	-0.12
ΔH_{gel}	-0.11	-0.61	-0.70*	-0.43	0.28	-0.72*	0.65*
T_o'	0.04	-0.52	-0.61	0.39	0.32	-0.71*	0.80*
T_p'	0.87*	0.82*	-0.06	-0.32	0.50	0.43	-0.13
T_c'	0.21	0.72*	0.29	0.21	-0.15	0.78*	-0.75*
ΔH_{ret}	0.16	0.37	0.27	-0.18	-0.56	0.17	-0.45
PV	-0.47	-0.56	-0.46	-0.06	0.70*	-0.58	0.16
HV	-0.44	-0.45	-0.38	0.03	0.75*	-0.48	0.10
BV	-0.49	-0.77*	-0.59	-0.27	0.47	-0.77*	0.31
FV	-0.43	-0.45	-0.53	-0.11	0.72*	-0.58	0.08
SV	-0.23	-0.05	-0.41	-0.07	0.76*	-0.37	-0.14
PT	-0.59	-0.28	0.34	0.37	0.37	0.00	-0.48
P_{Temp}	-0.47	-0.12	-0.44	-0.19	0.36	-0.19	-0.66*

* and ** indicated the significance at $P < 0.05$ and $P < 0.01$ level, respectively (n = 7). TSC, AAC, CPC, and TAAC are the total starch content, apparent amylose content, crude protein content, and total amino acid of ginkgo kernel flours, respectively. T_o, T_p, T_c, and ΔH_{gel} correspond to the onset temperature, peak temperature, conclusion temperature, and enthalpy of gelatinization of native flours, respectively. T_o', T_p', T_c', and ΔH_{ret} are the onset temperature, peak temperature, conclusion temperature, and enthalpy of gelatinization of retrogradation, respectively. PV, HV, BV, FV, SV, PT, and P_{Temp} are the peak viscosity, hot viscosity, breakdown viscosity (PV-HV), final viscosity, setback viscosity (FV-HV), peak time, and pasting temperature, respectively.

Supplementary Table S4. Pasting properties of kernel powders from seven gingko cultivars treated with water, DDT, and $AgNO_3$

Samples	PV(cP)	HV(cP)	BV (cP)	FV (cP)	SV (cP)	PT (min)	P_{Temp}(℃)
Powders in Water							
QXG	634.50±19.10^{f}	327.50±13.40^{g}	300.00±15.60^{d}	427.00±22.60^{f}	-192.30±15.60^{g}	4.70±0.10^{b}	82.10±0.20^{c}
DFZ	1162.00±19.80^{d}	810.50±17.70^{d}	340.50±17.70^{c}	1021.00±14.10^{c}	-48.00±20.50^{d}	5.10±0.10^{a}	82.90±0.10^{b}
DTH	1946.50±16.30^{a}	1446.00±17.00^{a}	489.50±14.80^{b}	2121.00±17.00^{a}	204.70±18.40^{a}	5.00±0.10^{ab}	82.80±0.10^{b}
FZ	1818.50±20.50^{b}	1255.50±12.00^{b}	555.00±19.80^{a}	2112.00±15.60^{a}	188.70±15.60^{b}	5.00±0.10^{ab}	83.80±0.10^{a}
ML	532.00±19.80^{g}	416.50±13.40^{f}	110.00±14.10^{e}	541.00±18.40^{e}	-40.30±4.90^{c}	4.80±0.10^{b}	82.90±0.10^{b}
DLY	972.50±13.40^{e}	673.50±16.30^{e}	286.50±14.80^{d}	836.50±21.90^{d}	-114.30±19.10^{f}	5.00±0.10^{ab}	83.10±0.10^{b}
LY	1657.50±13.40^{c}	1064.50±17.70^{c}	581.50±12.00^{a}	1598.50±17.70^{b}	-71.50±21.90^{e}	4.80±0.20^{b}	83.00±0.00^{b}
Powders in $AgNO_3$							
QXG	3457.00±17.00^{b}	1683.00±15.60^{bc}	1763.50±16.30^{b}	2434.00±18.40^{d}	-1011.50±14.80^{e}	4.50±0.30^{a}	79.40±0.40^{ab}
DFZ	2739.00±18.40^{f}	1455.00±18.40^{e}	1274.50±13.40^{f}	2142.00±14.10^{e}	-589.00±15.60^{c}	4.70±0.20^{a}	79.20±0.80^{ab}
DTH	3233.50±17.70^{d}	1772.00±12.70^{a}	1454.00±15.60^{d}	2631.00±15.60^{a}	-593.00±15.60^{c}	4.50±0.30^{a}	78.60±0.50^{b}
FZ	3328.50±17.70^{c}	1662.50±14.80^{c}	1656.50±16.30^{c}	2543.00±15.60^{b}	-769.50±24.70^{d}	4.40±0.10^{a}	79.40±0.30^{ab}
ML	2369.50±17.10^{g}	1375.50±17.70^{f}	984.50±13.40^{g}	2021.00±17.00^{f}	-333.50±21.90^{a}	4.60±0.20^{a}	80.10±0.40^{a}
DLY	2936.50±17.70^{e}	1524.00±15.60^{d}	1403.00±15.60^{e}	2496.00±12.70^{c}	-431.00±18.40^{b}	4.60±0.20^{a}	79.40±0.30^{ab}
LY	3591.00±12.70^{a}	1712.00±14.10^{b}	1866.00±17.00^{a}	2562.50±16.30^{b}	-1014.50±16.30^{e}	4.40±0.20^{a}	78.80±0.40^{b}
Powders in DTT							
QXG	1694.00±15.60^{d}	996.50±21.90^{e}	683.50±13.40^{a}	2301.50±14.80^{f}	595.50±11.50^{c}	5.20±0.00^{d}	83.80±0.20^{c}
DFZ	1845.00±18.40^{c}	1353.50±16.30^{c}	484.00±12.70^{b}	3157.50±16.30^{a}	1301.00±10.00^{a}	5.40±0.00^{bc}	83.60±0.20^{c}
DTH	2323.00±18.40^{a}	2151.50±12.00^{a}	165.00±15.60^{g}	2430.50±13.40^{d}	96.00±8.00^{f}	7.00±0.00^{a}	84.50±0.20^{b}
FZ	2219.00±17.00^{b}	2001.00±17.00^{b}	207.00±15.60^{f}	2364.50±12.00^{e}	132.00±10.00^{e}	6.90±0.10^{a}	85.30±0.20^{a}
ML	895.00±11.30^{e}	542.50±21.90^{f}	333.00±17.00^{d}	704.00±17.00^{g}	-195.00±8.00^{g}	4.80±0.00^{cd}	83.80±0.20^{c}
DLY	1722.00±15.60^{d}	1298.00±17.00^{d}	415.00±11.30^{c}	2892.00±15.60^{b}	1161.50±8.50^{b}	5.60±0.00^{b}	83.60±0.20^{c}
LY	2297.00±14.10^{a}	2023.50±17.70^{b}	263.50±10.60^{e}	2843.00±17.00^{c}	534.50±13.50^{d}	6.50±0.00^{a}	83.70±0.20^{c}

Data are given as means ± standard deviation (n = 3). Values in the same column with different letters were significantly different ($P < 0.05$). PV, HV, BV, FV, SV, PT, and P_{Temp} are described in the footnotes of Table S3.
